# Exploring Perception of Menopausal Symptoms and Social Support Seeking Behavior among Post-menopausal Women: A Qualitative Study

**DOI:** 10.31729/jnma.v63i2091.9222

**Published:** 2025-11-30

**Authors:** Pratiksha Paudel, Shristi Thapa, Bipsana Shrestha, Shrinkhala Shrestha

**Affiliations:** 1 Dhulikhel Hospital-Kathmandu University Hospital, Research and Development Division, Kavre, Nepal; 2 Kathmandu University School of Medical Sciences, Department of Public Health Programs, Kavre, Nepal; 3 Department of Health Services, Kathmandu, Nepal

**Keywords:** *menopausal symptoms*, *socio-ecological model*, *women*

## Abstract

**Introduction:**

Menopause is a natural phenomenon, however symptoms persist in many women. Geographical location and individual factors influence the perception and care-seeking behavior. There are virtually very limited studies exploring the perception and health seeking behavior of women in post menopause. Thus, this study aims to explore the perception of women regarding menopausal symptoms and individual, family, institutional and community level support for addressing the symptoms among post-menopausal women.

**Methods:**

An exploratory qualitative study design was conducted among the post menopausal women with symptoms residing in Dhulikhel Municipality. In-depth interviews were taken among 14 participants following a socio-ecological framework. The recorded interviews were transcribed and analyzed using deductive inductive thematic analysis approach.

**Results:**

Findings revealed the low level of awareness among women regarding knowledge and perception of menopausal symptoms. Furthermore, most of the women perceived menopause as a normal phase of life and didn’t recognize the symptoms. Limited awareness, past reliance on home births, and discomfort with male doctors reduced women’s use of health facilities. Community engagement varied some women shared experiences through local networks, while others avoided participation due to age, low literacy, or perceived irrelevance.

**Conclusions:**

The findings from the study highlight the need for culturally sensitive and gender-responsive interventions that strengthen health education, family involvement, community engagement, and empathetic healthcare to improve women’s well-being during and after menopause.

## INTRODUCTION

According to the World Health Organization, menopause is defined as permanent cessation of menstruation.^1^ The natural age of menopause occurs between 45 and 55 years for women worldwide.^2,3^

Menopausal period has the drastic hormonal changes which ultimately leads to permanent amenorrhea.^4^ These changes in hormonal balance results in symptoms like hot flashes, loss of bone mineral density that may cause long-term concern.^4^ Along with these symptoms women also develop risk to non-communicable diseases.^5^ Menopausal symptoms have effect on the quality of life; increased awareness and acquisition of coping strategies is the key for the management of symptoms.^6^

Menopause is a complex phenomenon and its experience is influenced by cultural norms, social factors, and knowledge. There is need for better understanding of social support. Thus, this study aims to explore the perception of women regarding menopausal symptoms for addressing the symptoms and social support and health care seeking behavior.

## METHODS

An exploratory qualitative study was conducted to explore the perception and care seeking behavior of post menopausal women in Nepal. Socio-ecological models were used to identify barriers and facilitators for women at individual, family, community and institutional level to seek social support for the management of the menopausal symptoms.^7^ A qualitative study was conducted to explore the perception and perceived social support at individual, family, community and institutional level that contributed to the menopausal symptoms. The goal of this approach was to further understand the perception of women, towards receiving support for identification and management of menopausal symptoms. This study was conducted at Dhulikhel municipality. Dhulikhel municipality lies in Kavrepalanchowk district situated 30 km southeast of Kathmandu valley. Dhulikhel municipality has an area of 54.62 sq. km with a population of 32026. Dhulikhel is a semi-urban area with 12 wards. Newar is a predominant ethnic group.^8^ The municipality is selected based on convenience and the feasibility to conduct study. The participants identified to be in post-menopausal stage were selected purposively and face-to-face in-depth interviews were carried out with them. Women who were residing in Dhulikhel municipality and those who didn’t have menstruation in the past 12 months were included. Similarly, those women who had hysterectomy, hearing impairment and women with mental disorders were excluded.

The participants for in-depth interview were selected purposively. The participants who had not had menstruation in the past 12 months were interviewed in order to explore their perceived knowledge and experience regarding menopausal symptoms and perceived family, community and institutional support. Interviews were stopped when no new information was obtained on the themes. A total of 14 in-depth interviews (IDIs) among post-menopausal women were conducted. A range of thematic issues were identified and code saturation was expected to reach at 14 interviews so the interview was stopped after reaching the code and meaning saturation.^9^ Interviews were carried out face-to-face and written consent were taken from all the participants and all the interviews were audio recorded on a mobile phone. The face-to-face IDI was conducted in Nepali language and the interview lasted from 20 to 45 minutes. Semi-structured IDI guidelines were used, which were developed using socio-ecological models. The goal of the interview was to explore the perception of women towards menopausal symptoms and familial, institutional and community support they seek for. An iterative process was used by discussing each interview shortly after it was completed and making suggestions for the future interviews, with subsequent interviews probing more deeply into themes emerging in earlier interviews. The IDIs were moderated by the investigator.

Audio recordings were updated daily in a password protected laptop for the back-up. The audio recorded interviews were transcribed verbatim in Nepali. The quality of all transcripts was ensured by cross-checking the transcripts with audio recordings. The transcripts were read multiple times before coding. The confidentiality of the participants was maintained by providing participant id. Finally, data analysis was carried out to generate themes and code from the data. The audio recordings were stored in the password locked computers where only authorized persons had access. For analysis, deductive inductive coding was used. Analysis was started with a set of predetermined codes as per our prior knowledge and then new codes were added after interviews. A thematic framework method, following Barun and Clarke’s analysis process was used for data analysis to identify themes related to perception regarding menopausal symptoms.^10^ The investigator read through transcripts several times to familiarize with the data. The text was then divided into meaningful units, such as phrases and quotes, and meaningful units were then coded. The condensed meaningful units were then abstracted and labeled codes by investigators. All the process was conducted manually with the research team.

This study obtained written permission from the institutional review committee i.e. Kathmandu University Institutional Review Committee (IRC no: 40/22). The data was collected from August to October 2022. Participants were informed about research details such as objectives, their role in study, the risk and benefits of participating in the study. Voluntary participation of the participants was assured and confidentiality of the information obtained from the participants was maintained.

## RESULTS

A total of 14 IDIs were conducted to explore the underlying perception of women towards menopausal symptoms and family, community and institutional level support for its management (Table 1). A socio-ecological model under the broad theme of individual, family, community and Institutional support that explores the perception and knowledge of women towards management of menopausal symptoms.

**The individual, family, institutional and community level support for addressing menopausal symptoms among postmenopausal women**

**1. Individual level understanding and perspective towards menopause**

The reasons relating to how women perceive menopausal symptoms and acknowledging the physiological changes as a woman passes through the stage.

**A. Knowledge regarding physical/physiological change during menopause**

Most of the women didn’t have any knowledge about the physical and psychological changes that occur during the menopause. Most of the women considered it as a normal phase that a woman goes through during her life and knew about it through family environment and communication.

Women believed that menopause was a natural process and could not happen if contracted from any disease. While some from their own experience shared that early menopause could be because of the use of contraception.

**Table 1:** Socio-demographic characteristics of the participants of IDI (n = 14).

CODE	Participants no.	Age	Marital Status	Education	Occupation
IDI_1	P1	58	Widowed	Illiterate	Housewife
IDI_2	P2	59	Married	Literate without formal education	Housewife
IDI_3	P3	56	Married	Literate without formal education	Housewife
IDI_4	P4	51	Married	Primary	Business
IDI_5	P5	44	Married	Illiterate	Agriculture
IDI_6	P6	45	Married	Literate without formal education	House wife
IDI_7	P7	48	Married	Literate without formal education	House wife
IDI_8	P8	56	Married	Illiterate	House wife
IDI_9	P9	60	Married	Illiterate	House wife
IDI_10	P10	50	Married	Illiterate	Agriculture
IDI_11	P11	60	Widowed	Illiterate	Agriculture
IDI_12	P12	54	Married	Primary	House wife
IDI_13	P13	43	Married	Secondary	House wife
IDI 14	P14	56	Married	Literate without formal education	House wife

IDI= In-Depth Interview.

*“Now... how should it feel, when I went for check-up, they had also told me this this.. might happen I might get anxious, have different thoughts and various other things.” (P6)*

*I didn’t think anyone will have any problem during menopause, rather I thought I would be happy if it just stopped. I never felt anything as such. I did not have any symptoms thus I thought others will also not have it. (P14)*

*“It’s like this, we had seen our mother go through the menopause, thus we guessed it would be the same way for us as well.” (P10)*

*“I don’t think menopause occurs because of some disease, I think after a certain age women enter menopause and it cannot occur earlier if anyone has disease.” (P8)*

**B. Effect of menopause in women’s life**

Most of the women felt relieved after menopause because after menopause they didn’t have to worry about being deprived of different freedoms that they had to go through only because of menstruation. Additionally, they indicated that they were rather happy being in their menopause because of various cultural norms and restrictions in menstruation.

*“In our home during menstruation we have strict restrictions. We are not allowed to go anywhere we want, touch anything and be involved in different rituals. My husband himself gets affected (deutaa chadne) if we do anything that is restricted. And now as menstruation has stopped I feel relieved.” (P7)*

*“As now after menopause I don’t need to worry about any restrictions during periods, I can take part in festivals without any doubt, I feel I am almost like a man now not worrying about all this.” (P12)*

**2. Knowledge and Experience about menopause and symptoms**

Knowledge and experience majorly explored women’s knowledge regarding menopausal symptoms irrespective of her experience.

**A. Knowledge regarding symptoms**

Many women were not aware about the occurrence of symptoms because of menopause. However, some women shared that as a woman enters into post-menopausal phase she develops increased risk of bone pain, cataract.

*“Currently I am having difficulty, it does not happen to everyone and even for me it will stop after sometime. I am not sure about it.” (P7)*

*“While going through menopause they say different women face different symptoms. Some have heavy sweating but as I have not experienced it I do not know much about it.” (P4)*

**B. Experience of symptoms**

Many women shared about experiencing various physical and somato-vegetative symptoms but were not aware that the symptoms could be because they were in post-menopausal stage. However, some women didn’t experience any symptoms thus they were not aware about it.

Some women shared that with women entering into the postmenopausal phase she has increased risk of bone pain.

*“I did not have these symptoms earlier, but now I do not have good sleep, I also have sweating in my body even during the night and have pain in my knees and fingers.” (P8)*

*“Sometimes it’s very difficult for me to fall asleep. I also have a bone problem in my knee. I also went for a check-up. (Nasaa chepeko)” (P3)*

*“....in this case may be because of menopause or If I do not know if it is anything else but I have extreme burning sensation and sweating, also my body feels exhausted (jiu phatryaak phutruk bhaera galne.) Also I had heavy bleeding during my last menstruation.” (P6)*

*I am having severe back pain. Also, earlier when I had regular menstruation I used to feel my lower abdomen is light but now as I have not had my menstruation for such a long period I feel like something heavy in my lower abdomen. I also have joint and breast pain.” (P13)*

**C. Experience of menopause**

The experience regarding menopause differed between women, some women complained about having heavy and irregular bleeding during menopause whereas for some it was as usual. However, one participant complained of not having menstruation since the use of contraceptive device (IUCD).

*“When my menstruation was about to stop I had irregular menstruation cycle, sometimes in 5-6 months, when I had menstruation in 5-6 months bleeding was less, sometimes my cycles were as short as 15 days but it was only spotting than I again menstruated in 4 months, 5 months, 6 months and it really stopped.”(P2)*

*“It has been five years since my last menstruation. Once while I was using depo, then I stopped using it, then after putting this (IUCD) I had menstruation once in between and then after I did not have my periods. I went to doctors for check-up too, they said it has not affected me inside.”(P5)*

*“It has been three years since my last menstruation. Earlier I had my periods every month and then it started to become irregular, once in 2 months, 3-4 months 5-6 months and a year. Later when I had my menstruation in 6 months and in a year I had very heavy bleeding, it was like I was urinating. I was really afraid and even thought of going to hospital for a check-up. Then again I thought everyone complains of having similar problems so I do not need to go.” (P14)*

**3. Family’s understanding about menopause and their roles**

Family’s understanding is majorly about how women perceives the role of family if women experiences menopausal symptoms.

**A. Role of family to manage symptoms of menopause**

Most of the women were confident that their family would take care of them in need. The presence of a husband had a huge impact in how women considered family’s support.

Some of the women shared that they were capable of handling different household chores even if they are sick and felt it was only their responsibility to look over it.

*“Um.. I don’t feel anything as such as I do not have family, I have one daughter she is also already married, I live alone in the house so I don’t feel anything as such.” (P1)*

*“My husband is also not very well but he goes for work but cannot work very well but I have sons who stay with us and look after me if I am sick or need any support.” (P11)*

**B. Type of support from family**

Most of the women shared that their family would support them if they had to visit the hospital.

*“Till now I have not felt that way. I have never felt like I cannot cook, wash my clothes or bring a sack of grass and my family needs to help me with it.” (P2)*

*“When I am sick and unable, I would want my family to help me with household work. To think about my pain and behave well with me. I often think why they won’t understand me and help me.” (P5)*

*“... Now from family all kinds of help, when I am unable, sick and in trouble, family, husband and children should help me by taking me to the hospital or by bringing me medicine or other such things. (P3)*

*“.... During that time we would want family members to look after us by cooking something, giving us time to rest but in many cases me might or might not get such support.”*

**4. Role of Health care facilities**

Health Institutions can play a major role in identifying and treating menopausal symptoms of women. However, many women are not aware about the symptoms and visit gynecologists only in extreme cases.

**A. Perception towards visiting health care facility**

Many women have never visited health care facility for reproductive health check-up and didn’t feel the need to visit the health care facility

*“I have never been to a hospital for such (menopausal, reproductive health) check-ups. I have four children. All of them I gave birth at home and also I didn’t go to the hospital for a pregnancy check-up. I didn’t do anything as I was not aware of such things. Also another thing is my mom and dad died when I was very young and no one taught me such things.” (P3)*

*“Earlier in our days I thought it was okay to not go for checkups but nowadays because of our eating patterns and lifestyle I think if we do not go to hospitals earlier than it might create more difficulty.” (P9)*

**B. Role of health professionals**

Most of the women shared that they would be willing to visit the hospital if the health care professional respected them, treated them well and did proper diagnosis and provided with the right medicine.

However, some of the women felt awkward if they were checked-up by male doctors and preferred going to hospital if they were treated with female doctors.

*“I would want the doctor to check me thoroughly, treat me well. Some doctors talk very politely while some are rude, then we also get angry by such behaviors sometimes. (P3)*

*“When we go to hospital we would want to check on time most of the time. Doctors are somewhere and we need to wait for a long time.” (P8)*

**5. Community level support and programs**

Many women are in close connection with the community and community can have many various impacts on women’s health and health seeking behavior.

**A. Social networking**

Many women were engaged in different social networking and felt heard in such groups. Whereas some women felt isolated and unwilling to share about their problems, especially health-related problems.

*“I am not involved in any such networking but when I am having problems we have our relatives around and we sit and talk about it. We also talk about health relatedprograms.”(P14)*

*“Yes I do talk about it in a community with neighbors around. We talk about problems in family and health and they provide me with consolation. When I was planning to have the operation I talked with my neighbors and they gave me suggestions.” (P5)*

*“As we do not have any such official networking groups, there are no such groups till now where we sit and talk about women’s health.” (P13)*

**B. Community based health related programs**

Many women were aware about different community based health related education programs but were unwilling to attend and participate in it because of their education status and old age.

*“It’s like this regarding an awareness program when last year we talked about doing such a program under my supervision for women for uterine prolapse and we conducted it as well so what happened is we had around 200 targets for check-up in that we could not even collect 50.” (P13)*

*“I don’t think I will be able to participate in such programs because I think I am already very old, I have never been to school. I just do labor work and eat so that’s all it is. (P11)*

*“It’s good to know about different things, if we know we can share it with others and also maybe we are having problems because we didn’t know. Information is changing everyday if today it is one thing than tomorrow it is another even though we are old we would want to know and learn about different things.”(P7)*

## DISCUSSION

Our study findings show that women’s experiences of menopause are influenced by individual perceptions, family support, health institutions, and community networks. Most women viewed menopause as a natural life stage rather than a health issue and lacked knowledge about its physical and psychological changes. Their understanding came mainly from family observations and informal discussions, not formal education. Although many experienced symptoms like hot flashes, joint pain, and sleep problems, they often attributed them to aging or general ill health. Family members, especially husbands and children, played key roles in providing emotional and practical support, while women living alone often felt isolated. Limited awareness, past reliance on home births, and discomfort with male doctors reduced women’s use of health facilities. Community engagement varied some women shared experiences through local networks, while others avoided participation due to age, low literacy, or perceived irrelevance. Most women in our study lacked knowledge about the physical and psychological changes that occur during menopause, as they perceived it as a normal stage in a woman’s life. These findings align with studies among Malaysian women, who viewed menopause as a natural part of aging and accepted it as an inevitable stage in life.^11-13^

Most women in our study felt relieved and satisfied after reaching menopause, as it freed them from the cultural and religious restrictions associated with menstruation. They no longer worried about being excluded from household activities, religious rituals, or social events, which often restrict women during menstruation in many Nepali communities. This newfound freedom allowed them to participate more actively in daily and cultural life, enhancing their sense of autonomy and well-being. Several studies report similar findings, showing that women view menopause positively as a period of liberation from taboos, physical discomfort, and reproductive responsibilities.^11,^ ^14-16^ These findings highlight how sociocultural norms strongly shape women’s experiences of menopause.

Additionally, our study found that women’s knowledge and experiences of menopause and its symptoms varied widely. Many women did not recognize that their symptoms were related to menopause. Several women reported experiencing physical and somato-vegetative symptoms but did not associate them with the post-menopausal stage. Experiences also differed between women: some reported heavy or irregular bleeding, while others experienced menopause without noticeable changes. These findings align with the study conducted in East Coast Malaysian women, where participants’ most commonly reported symptoms were somatic and psychological.^11^ On the contrary, several studies in Asian and Western countries have reported a higher occurrence of vasomotor and urogenital symptoms than our study participants indicated.^15,17,18^

Our study found that family support greatly influenced how women managed menopausal symptoms. Most women felt confident that their families, especially their husbands, would care for them when needed and help them visit health facilities or seek medical care. In contrast, a study on menopause knowledge, attitudes, and experiences among women in Saudi Arabia reported that participants felt isolated and disconnected during perimenopause, indicating limited social support.^19^. These findings highlight that while family support can provide emotional and practical assistance, traditional norms may limit women’s ability to prioritize their own health during menopause. Additionally, our study found that although health institutions play a vital role in identifying and managing menopausal symptoms, many women rarely sought medical care unless symptoms became severe and never visited a health facility for reproductive health check-ups which is similar to Malaysian women study where none of the participants sought treatment for their menopausal symptoms.^11^ However, they expressed willingness to seek care if health professionals treated them respectfully, provided proper diagnoses, and prescribed appropriate medications.

Moreover, our study also found that community connections strongly influenced women’s health awareness and help-seeking during menopause. Many women participated in social networks and felt supported, while others felt isolated and reluctant to discuss private health issues. Although many knew about community health programs, few participated due to low literacy, older age, or perceived irrelevance. These findings highlight the need for inclusive, age-friendly, and culturally sensitive community initiatives to promote open discussions and support for menopausal women.

This study has several strengths that enhance its credibility and relevance. By using the Socio-ecological model we applied a structured approach to explore perception of menopausal symptoms. Phenomenological in-depth interviews captured rich, lived experiences and contextual factors. Conducting face-to-face interviews improved data quality by fostering rapport and allowing for the observation of nonverbal cues. However, our findings are limited, which may affect the broader applicability of our findings. Conducted in a single semi-urban municipality, thus the results may not be generalizable to other regions with different contexts. At last, Social desirability bias may have influenced responses of the participants. This could have limited the depth and accuracy of insights into the actual health seeking behavior.

## CONCLUSION

This study shows that postmenopausal women’s understanding and management of menopause are shaped by individual, family, institutional, and community support. Most women viewed menopause as a natural stage but lacked knowledge about its symptoms. While some faced discomfort and uncertainty, others felt relief from menstruation-related restrictions. Family support, particularly from husbands and children, played a key role in coping, although traditional gender roles often limited rest during illness. Many women sought medical care only when symptoms became severe and preferred respectful, female healthcare providers. Community networks provided emotional support, but participation in health programs was low due to age, literacy, and perceived irrelevance. These findings highlight the need for culturally sensitive and gender-responsive interventions that strengthen health education, family involvement, community engagement, and empathetic healthcare to improve women’s well-being during and after menopause.

## Data Availability

The data are available from the corresponding author upon reasonable request.
